# Analysis of the Antennal Transcriptome and Identification of Tissue-specific Expression of Olfactory-related Genes in *Micromelalopha troglodyta* (Lepidoptera: Notodontidae)

**DOI:** 10.1093/jisesa/ieac056

**Published:** 2022-09-27

**Authors:** Yu Zhang, Kai Feng, Ruolan Mei, Wei Li, Fang Tang

**Affiliations:** Co-Innovation Center for the Sustainable Forestry in Southern China, Nanjing Forestry University, Nanjing 210037, China; College of Forestry, Nanjing Forestry University, Nanjing 210037, China; Co-Innovation Center for the Sustainable Forestry in Southern China, Nanjing Forestry University, Nanjing 210037, China; College of Forestry, Nanjing Forestry University, Nanjing 210037, China; Co-Innovation Center for the Sustainable Forestry in Southern China, Nanjing Forestry University, Nanjing 210037, China; College of Forestry, Nanjing Forestry University, Nanjing 210037, China; College of Life Sciences, Yangtze University, Jingzhou 434025, Hubei Province, China; Co-Innovation Center for the Sustainable Forestry in Southern China, Nanjing Forestry University, Nanjing 210037, China; College of Forestry, Nanjing Forestry University, Nanjing 210037, China

**Keywords:** odorant receptor, odorant-binding protein, chemosensory protein, ionotropic receptor, sensory neuron membrane protein

## Abstract

*Micromelalopha troglodyta* (Graeser) has been one of the most serious pests on poplars in China. We used Illumina HiSeq 2000 sequencing to construct an antennal transcriptome and identify olfactory-related genes. In total, 142 transcripts were identified, including 74 odorant receptors (ORs), 32 odorant-binding proteins (OBPs), 13 chemosensory proteins (CSPs), 20 ionotropic receptors (IRs), and 3 sensory neuron membrane proteins (SNMPs). The genetic relationships were obtained by the phylogenetic tree, and the tissue-specific expression of important olfactory-related genes was determined by quantitative real-time PCR (qRT–PCR). The results showed that most of these genes are abundantly expressed in the antennae and head. In most insects, olfaction plays a key role in foraging, host localization, and searching for mates. Our research lays the foundation for future research on the molecular mechanism of the olfactory system in *M. troglodyta*. In addition, this study provides a theoretical basis for exploring the relationship between *M. troglodyta* and their host plants, and for the biological control of *M. troglodyta* using olfactory receptor as targets.

Complex chemical sensory mechanisms are essential for the survival and reproduction of insects. The accurate olfactory sensory system plays a role in many insect behaviors, such as searching for food, finding mates, choosing spawning sites, and avoiding natural enemies (Brigaud et al. 2009, Zhou 2010, Lu et al. 2015). As an important part of the tissues insects use to sense and recognize odors, antennas are distributed with a large number of olfactory-related proteins. The proteins related to the insect olfactory system include: odorant receptors (ORs), odorant-binding proteins (OBPs), chemosensory proteins (CSPs), ionotropic receptors (IRs), odorant degrading enzymes (ODEs), and sensory neuron membrane proteins (SNMPs) (Leal, 2005).

The OR genes of insects are highly diverse, and the numbers between different species vary greatly; most of them posses between 50 and 100 (Nei et al. 2008). The first insect ORs were identified in *Drosophila melanogaster* Meigen by screening genomic data (Qiao et al. 2008). Insect odorant receptors (ORs) are a class of transmembrane proteins that form an ion channel on the dendritic membrane of neurons. ORs are mainly divided into two types. One is the conventional odorant receptor (specific OR), which has low homology among different insects. The other receptor is an atypical common receptor now known as Orco, which is highly conserved among different insects (Vosshall and Hansson 2011). Some studies have suggested that Orco acts as a partner of odorant receptors and works together with them (Grosse-Wilde et al. 2011, Stengl and Funk 2013).

Odorant-binding proteins (OBPs) are a class of spherical hydrophilic proteins located in the lymph fluid of insect antennae. OBPs can be divided into three categories: sex pheromone binding proteins (PBPs), general odorant binding proteins (GOBP), and antenna bind proteins (ABPx) (Vogt and Riddiford 1981, Vogt et al. 1991, Krieger et al. 1996). It has been reported that PBPs, as members of the sex pheromone identifying protein family, mainly bind to insect sex pheromones, while GOBPs are involved in identifying common odorants and sex pheromones (Grosse-Wilde et al. 2006, Wang et al. 2022). Wang et al. (2020) found that two potential PBPs (BgerOBP26 and BgerOBP40) were identified in *Blattella germanica (*L.). OBPs, as important participants in insect olfactory behavior, combine with lipophilic odorants in lymph fluid to form complexes, and transport them to the dendritic membrane of sensory neurons. The combination with odorants is the first step to start odorant perception. In addition, other proteins such as chemosensory proteins (CSPs), ionotropic receptors, odorant degrading enzymes(ODEs), sensory neuron membrane proteins (SNMPs), and ionic receptors (IR) are also involved in the process of odorant perception (Scott et al. 2001).

There are obvious differences between CSPs and OBP in sequence, structure, expression profile, and so on (Pieimbon et al. 2000). Unlike OBP, which is highly enriched in antennae, CSPs have a wide range of tissue expression profiles (Wang et al. 2005, Pelosi et al. 2006). ODEs are considered to play key roles in odorant inactivation to maintain the odorant receptor sensitivity of insects. Some members of carboxylesterase (CXE) are a major subfamily of ODEs. He et al. (2020) found that a ubiquitous expression esterase SexiCXE11 may be partly involved with olfaction. In addition, SNMPs are the important olfactory functional proteins in insects. Liu et al. (2014) found that *SexiSNMPs* have dual functions in receptive pheromone and common odor recognition in *Spodoptera exigua* (Hübner). Furthermore, the ionotropic glutamate receptor (iGluR) family were named ionic receptors (IR) in *D. melanogaster* (Benton et al., 2009). 12 IRs genes have been identified in the antennae of *Spodoptera litura* (Fabricius) (Olivier et al., 2011);15 IR_S_ gene have been found in the antenna of *Cydia pomonella* (L.) (Bengtsson et al., 2012).

*M. troglodyta* larvae feed on the leaves of poplars and are the main pests that endanger poplars. There have been outbreaks in the northeast, northwest, central plains, and south central China. Especially in the case of a single tree species, the leaves are often completely eaten during pest outbreaks, which not only affects the normal growth of poplars and causes economic losses but also destroys the ecological environment and restricts the development of forestry (Tang et al. 2009).

Olfaction plays an important role in life activities such as the feeding, mating, and positioning of *M. troglodyta*. As an herbivorous insect, its sensitivity and binding effect to plant volatiles can improve its selectivity and adaptability to host plants to enhance its invasion ability and lead to more serious harm. Although extensive studies have been carried out on the molecular mechanism of olfaction and the identification of chemical sensory genes in many Lepidoptera species (Elfekih et al. 2016), they may not be applicable to *M. troglodyta*. Therefore, the identification of olfactory genes is very important to clarify the molecular mechanism of olfaction and verify the unique olfactory receptors in *M. troglodyta*. However, there is no report on the olfactory repertoire and its tissue-specific expression in *M. troglodyta* in China or elsewhere. In this study, the transcriptome of the *M. troglodyta* antenna was analyzed, important olfactory-related genes (ORs, OBPs, SNMPs, IRs, and CSPs) were identified, and phylogenetic trees were used to evaluate their phylogenetic relationships with other species. In addition, the tissue-specific distribution of all olfactory-related genes was determined by qRT–PCR. These results will lay the foundation for clarifying the molecular mechanism of olfactory perception in the future. In addition, understanding olfactory behavior is essential for developing eco-friendly control strategies. For example, the combination of insect repellents or attractants is used to regulate the number of pests and natural enemies. Additionally, this study also provides a theoretical basis for exploring the relationship between *M. troglodyta* and host plants, which is important for the biological control of *M. troglodyta* using olfactory receptors as targets.

## Materials and Methods

### Experimental Insect Rearing and Sample Preparation

*M. troglodyta* population was collected from poplar in Nanjing and brought back to the laboratory for feeding. The larvae were placed in the incubator under the conditions of 26 ± 1°C, 70–80% RH, and a photoperiod of 16:8 (L:D) h, and fresh poplar leaves were collected daily as food.

The antennae, heads (without antennae), cuticles, midguts, forewings, and hindwings were dissected from adults, and then frozen in liquid nitrogen and stored in freezer at −80℃ for subsequent RNA extraction. Antennae were used for subsequent transcriptome sequencing. All tissues were used for expression profiling.

### RNA Isolation and Sequencing

Around 10 mg of tissues (mixed antennae) were extracted by *AG RNAex Pro* Reagent (AG21102, Accurate Biotechnology, Hunan, Co., Ltd). The extracted RNA was first analyzed by gel electrophoresis on 1% (w/ V) agarose gel to detect the integrity of RNA, and then the concentration of RNA was measured by spectrophotometer (Eppendorf Bio Spectrometer). Solexa sequencing using an Illumina HiSeq 2000 was performed by Shenzhen Huada Gene Research Institute.

### Sequence Assembly and Annotation

Trinity was used to perform the de novo assembly and eliminate duplicates. Then, Tgicl was used when the transcripts were aggregated into unigenes. The unigenes were blasted to compare with five databases (Nt, Nr, COG, KEGG, and SwissProt) to obtain the annotation results and the gene function was annotated from GO database using Blast2GO (Conesa et al. 2005).

### Phylogenetic Analysis

Phylogenetic trees were built based on amino acid sequence alignment of the candidate genes from *M. troglodyta* and those of other insects' species using ClustalX2.0. The phylogenetic tree was constructed using the Neighbor joining method (NJ), and the P-distances modeling and a pairwise deletion of gaps were performed by the MEGA X software package. The reliability of the tree structure and node support was evaluated by bootstrap analysis with 1,000 replicates (Tamura et al. 2013).

### Tissue-specific Expression of Olfactory-related Genes in *M. troglodyta*

The cDNA concentration of the tissues was adjusted to 50 μg/ml and the whole reaction preparations were carried out on ice. The primers for qRT–PCR were designed by online software (https://sg.idtdna.com/scitools/Applications/RealTimePCR/) (Table 1). qRT–PCR was performed with an ABI 7500 (Applied Biosystems) using SYBR Premix Ex Taq (Tli RNaseH Plus) (Takara, Japan). *β-actin* was used as reference gene. The melting curve was analyzed to evaluate the specificity and sensitivity of these primers, and LinReg PCR software (Version: September 2014) was used to examine the amplification efficiency of each gene. Each PCR system was set to 20 μl, including 10.0 μl SYBR premier ex Taq, 0.4 μl Rox II, 0.4 μl forward and reverse primer (10 mm), 2 μl sample cDNA (100 ng), and 6.8 μl double distilled water. qRT–PCR was divided into three stages. First, predenaturation was maintained at 95°C for 30 s, then 95°C for 5 s, 60°C for 34 s and 40 cycles at this stage, and finally 95°C for 15 s, 50°C for 1 min, 95°C for 15 s. At the end of the reaction, the amplification curve and the melting curve were confirmed. All experiments were performed four repetitions. The relative expression level of *M. troglodyta* mRNA was calculated by the 2^−ΔΔCt^ method (Giulietti et al. 2001).

**Table 1.** Primers of olfactory-related genes for qRT–PCR in *M. troglodyta*

Genbank	Primers	Sequence (5ʹ–3ʹ)
GU262991 (β-actin)	F R	GCGGCGCGACTCACCGACTAC GGGAAGAGAGCCTCAGGGCAAC
*MtroOR27*	F R	GGCTACCCTACATTGACTTTACC GTGCCGCACCCATACTATTT
*MtroOR29*	F R	GAAGACTTGCCAGAGACCTTATAC GCCAGAACCAAGAGGACTATTT
*MtroOR7*	F R	GGTAGGAAAGTCCGTTGG ATCTATGTGGGCTGTGGG
*MtroOR1*	F R	AGGTAAACGTGTTCAGTGAGAG AGTTCAAGAGACATCTGCGTATC
*MtroOR10*	F R	GAGAAGCGAGAGGTTGGTAAAG CGAGTGTGCAACAGAGAAGATA
*MtroOR13*	F R	CGGAGGTTTGGCTTAGACTATT GACGTAGTAGTGGAACGGAATG
*MtroOR19*	F R	CTCACGGCATCCTATTCTCTATG GCATGGCTCTCTCTCTTCTATG
*MtroOR16*	F R	ACGTTGCCGTATCCAGAAATAC TACCACGCCTCCTCCATAAA
*MtroOBP9*	F R	CATCATGCAGAGAGCGTACTT ACAGATGAGCAGAAGGAGAAAC
*MtroOBP6*	F R	GAACTCGAAGAACTCCTCCATC AGCCGACATGCACATCAT
*MtroOBP7*	F R	CTGCAAAGATGTCGCAAACAA TGAAACTCTTCGGGTCGTAATC
*MtroOBP1*	F R	TGCCCGTATAGTGGGTTTA TGGCGCTGATTGAGACAA
*MtroOBP31*	F R	TATACTTTCGATCATCTTCACC GCTATCTCCGCCTTTCTC
*MtroOBP12*	F R	AGAGCACCATGGCACATTTA GAAAGAGAGGAGATCGTCAACC
*MtroOBP14*	F R	GAGACGCCAAGAAGGAGG CAACATGCCAAGGTCAGC
*MtroOBP15*	F R	GAGAAATATGGGTCGGCTTCTTA GGGCTATAGAGTCGTAGGTATCA
*MtroCSP1*	F R	CGTACTTGTCGGTGTACTTCTC GGACACATCTGCCTGTCATTA
*MtroCSP2*	F R	ATATCCCGATGGTGGTGTTATG CCTTCACTGGTCTGTGATACTG
*MtroCSP3*	F R	ATTCTCCGCACTACCGTTTAC GGACGATTGGGTCAAACTAGAA
*MtroCSP4*	F R	GGCGAAGAACTGAAAGGACATA TCAGGTGCTTCATCACTTTCTC
*MtroCSP6*	F R	GTTGGTGGTCGCCACTATAA CCGCATACATCATCCGTTCT
*MtroCSP7*	F R	CGCCAAACCTGAATCAAACC CCGACTAGTACCAGCCATAGTA
*MtroCSP8*	F R	CGCAGTTCCTCCTCGTATTT GATCAAGCACCTCATCAACAAC
*MtroCSP9*	F R	TGCCAGCGCTCGAATAAA CAATAGACGTATCCCGCATCTG
*MtroIR1*	F R	GACGACGAACTGTCGATGAA CGCCAGAACCCTGTCAATAA
*MtroIR6*	F R	CTCTCCATGAACCTAGCGAATG GATGACCCAATGAACGGAAGA
*MtroIR7*	F R	CCCTACCTTCCCGTCATAAAC GGTCTTGTAACAGTGGTCTCTC
*MtroIR8*	F R	GTTCCGGTCTCATGGTCTTTAG CTACTGACGCGGCCATTATT
*MtroIR9*	F R	GCGACGAGCTATACTACCTAAAC TAACACTGCTCCTTTCCTAACC
*MtroIR10*	F R	GGCATGGTGGTAGATCATAGAG CGTCTCATTGCTGTCGGTAATA
*MtroIR11*	F R	CGAGCTATGGAAAGGGCTATG TGGCAATTCTCTGGGCTATG
*MtroIR12*	F R	ACTAGTAGGCGCTGTGTAAATG GTCCAGCCGTTTCAGAGATT
*MtroSNMP1*	F R	GACCCTGAGGTTCAGAAGAATG CTACCATAGGTGTGCCAGTTATC
*MtroSNMP2*	F R	CTACGATGCCCGCTACTAATAAA GATGGGTCAAGACTCGGATTAC

### Data Analysis

Data collected from these assays were subjected to analysis of variance using InStat software (GraphPad, San Diego, CA). The statistical significance of multiple sample comparisons was calculated using a one-way analysis of variance followed by Tukey’s multiple comparisons. A value of *P* < 0.05 was considered statistically significant.

## Results

### Transcriptome Sequencing and Sequence Assembly

In total, we obtained 59,076,354 clean reads from *M. troglodyta* antennal transcriptome. The percentages of clean reads with Q20 and Q30 quality scores were 98.65 and 95.56%, respectively. 101,597 transcripts were obtained by using Trinity, with an N50 of 1,587 bp, an average length of 740 bp, and a total length of 75,206,425 bp. 41,300 unigenes were selected from the above transcripts with a mean length of 1,092 bp and an N50 length of 2,118 bp. After eliminating repeated and short-length sequences, 41,300 Nr unigenes were annotated against five public databases using similarity searching. In total, 23,405 (56.67%) genes were annotated in the NR database; 16,677 (40.38%) genes were annotated in the Swiss-Prot database; 117,744 (42.96%) genes were annotated in the KEGG database; 8,790 (21.28%) genes were annotated in the KOG database; 2,822 (6.83%) genes were annotated in the GO database. In addition, 14,537 (35.2%) genes were not annotated with any database.

### Functional Annotation of the *M. troglodyta* Transcriptome

**Fig. 1.**
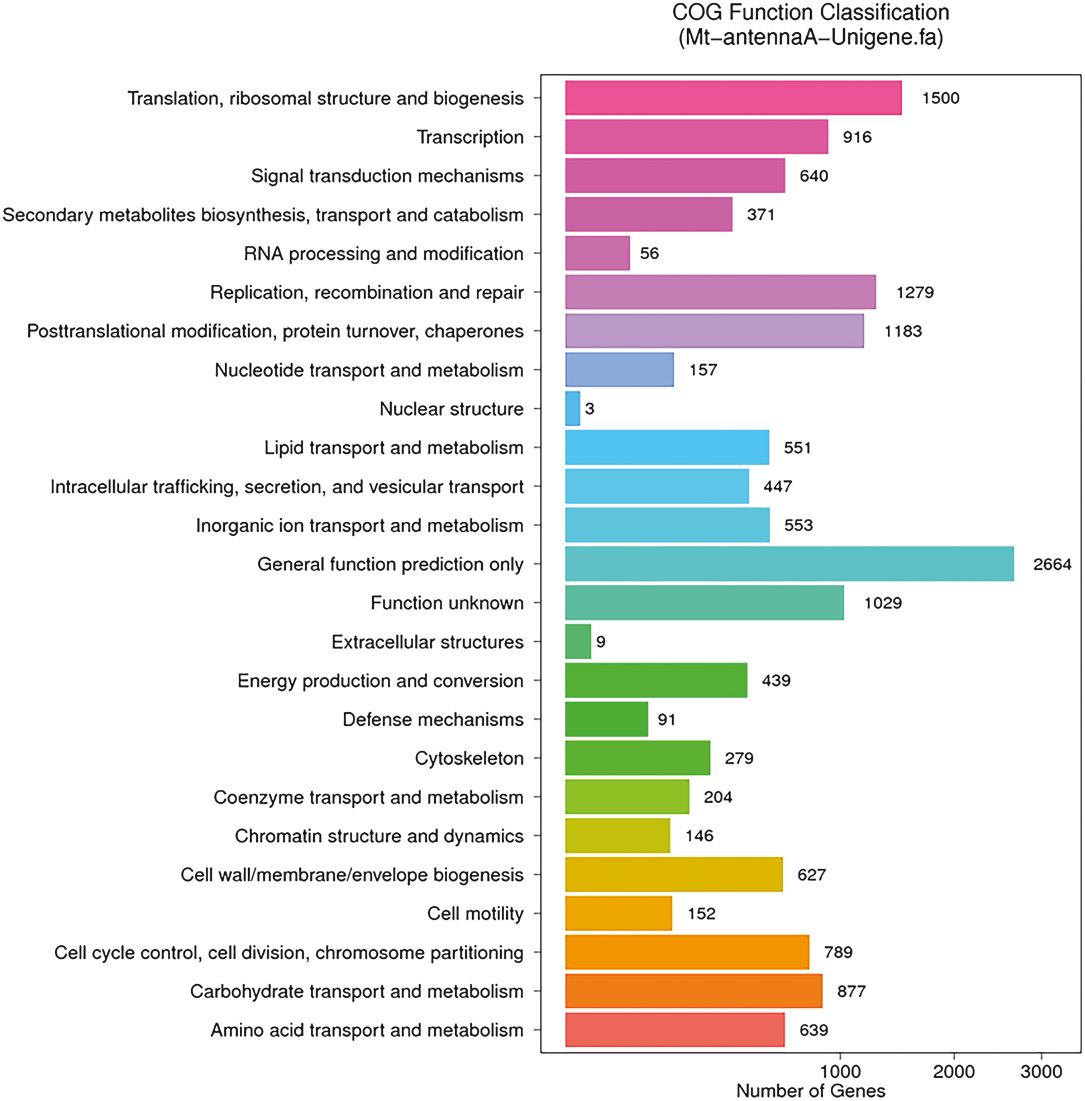
Clusters of Orthologous Groups (COG) classification analysis of *M. troglodyta* antennal genes.

The greatest number of unigene matches was for insect genomes, and *Amyelois transitella* (Walker) (22.98%), *Bombyx mori* L. (19.61%), *Papilio xuthus* L. (9.17%), and *Papilio machaon* L. (8.45%) accounted for the top 4 unigene matches based on the Nr annotations. The remaining 39.79% of the sequences showed good homology with those of other insects (Supp Fig. S1 [online only]). In total, 8,790 sequences were subjected to COG classification, and they were divided into 25 COG groups using WebMGA, with an E-value cut-off of 1e^−5^. This may be related to the fact that there is still currently little data on *M. troglodyta* in the COG database. The unigenes were annotated to the COG database and their possible functions were predicted. From the 25 COG categories, the cluster for ‘General function prediction only’ (2,664) was the largest group, followed by the ‘Translation, ribosomal structure and biogenesis’ (1,500) group. The ‘Nuclear structure’ (3) and ‘Extracellular structures’ (9) groups were the smallest classes (Fig. 1).

### Gene Ontology Annotation

**Fig. 2.**
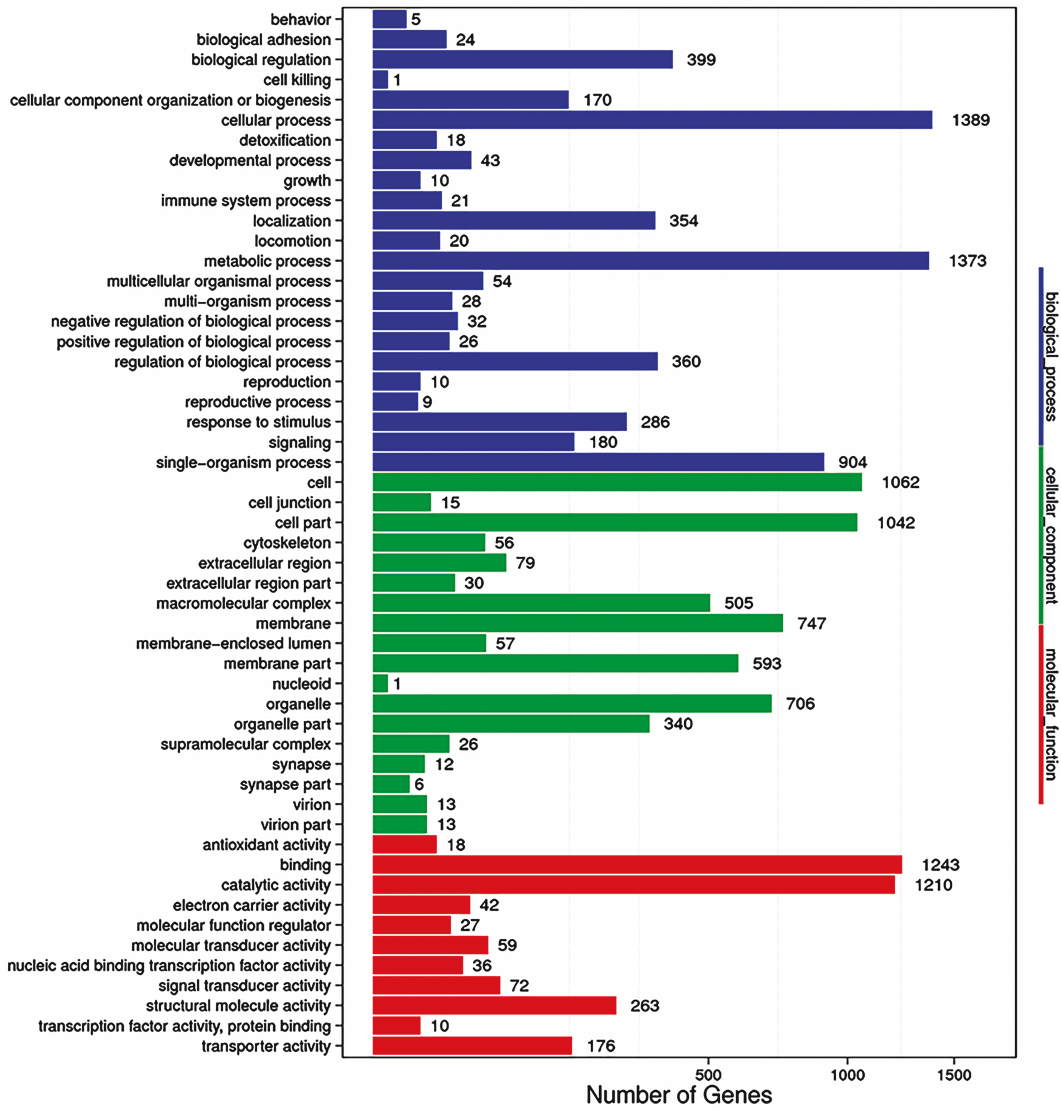
Gene Ontology (GO) classification analysis of *M. troglodyta* antennal genes.

The GO database is commonly used for gene functional annotation (Ashburner et al. 2000). Using Blast2Go software, the transcriptome of *M. troglodyta* was successfully mapped to the three main functional processes, which comprise 52 GO terms. In other words, according to the GO gene functional classification system, 2,822 unigenes were divided into the three major functional ontologies, biological process (BP), cellular component (CC), and molecular function (MF). In the category of biological processes, the main subcategories were cellular process (1,389) and metabolic process (1,373), followed by single-organism process (904). For the cellular component category, cell (1,062), cells parts (1,042), and membrane (747) were the most highly represented. In terms of molecular function, binding (1,243) and catalytic activity (1,210) were highly enriched. Nevertheless, in all three main categories, few genes were assigned to cell killing, nucleoid, behavior, and synapse part (Fig. 2).

### Identification and Tissue-specific Expression of Olfactory-related Genes

Through the analysis of the antennal transcriptome data of *M. troglodyta*, a total of 142 olfactory-related genes were identified, including 74 OR genes, 32 OBP genes, 13 CSP genes, 20 IR genes, and 3 SNMP genes. We downloaded the OR, OBP, CSP, IR, and SNMP gene sequences of other insects on NCBI, 5 phylogenetic trees were successfully constructed by the neighbor-joining (NJ) method using MEGA X. At the same time, we selected some genes to determine the tissue expression pattern.

### Identification and Tissue Expression Profiles of Putative Odorant Receptor (OR) Genes from *M. troglodyta*

**Fig. 3.**
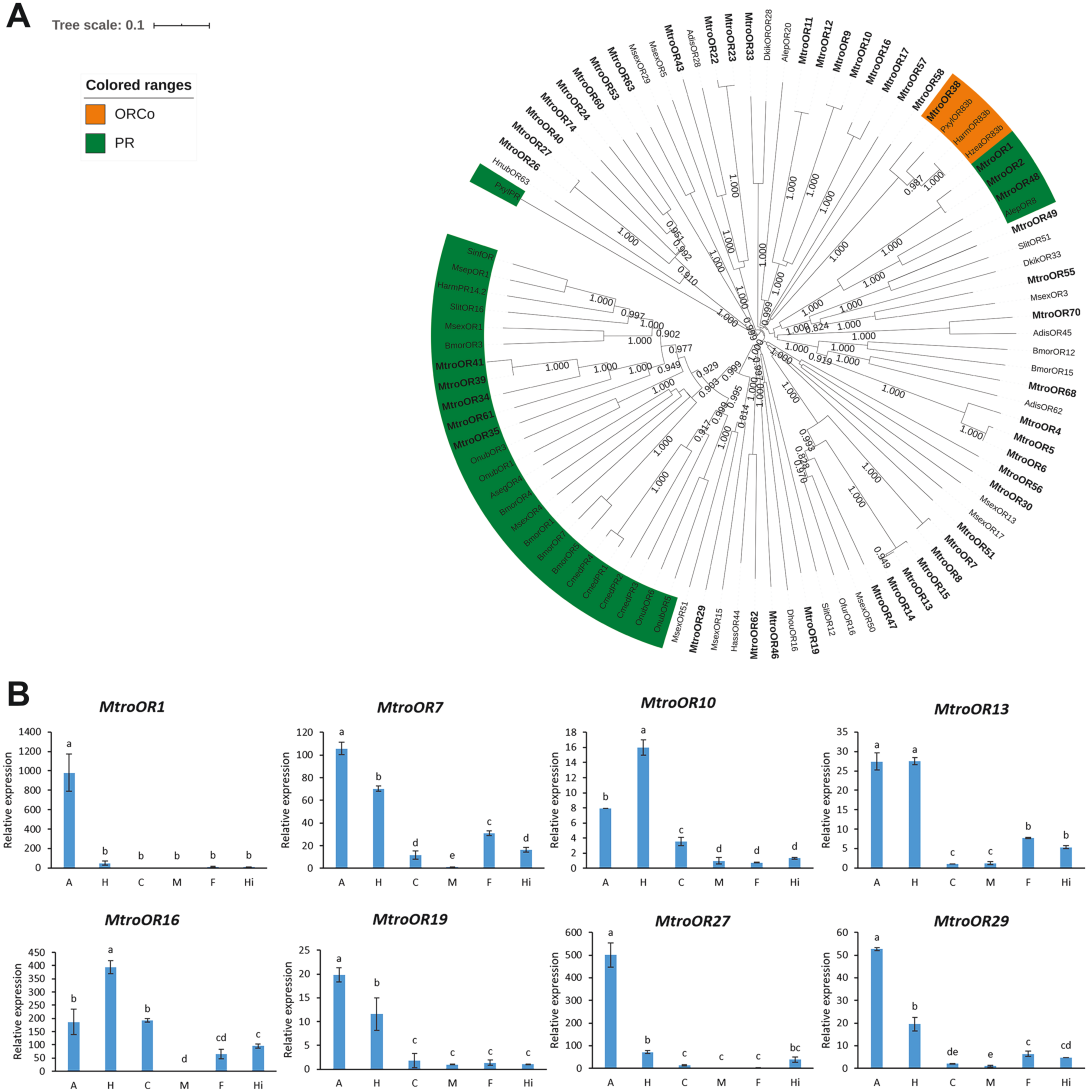
Phylogenetic analysis of ORs and the tissue-specific expression of ORs in *M. troglodyta*. (A) The phylogenetic tree was generated using MEGA X on the basis of ClustalW alignments. Branch numbers represent bootstrap values (1,000 replicates). *Bombyx mori, Bmor; Dendrolimus kikuchii, Dkik; Manduca sexta, Msex; Spodoptera litura, Slit; Athetis dissimilis, Adis; Helicoverpa assulta, Hass; Athetis lepigone, Alep; Plutella xylostella, Pxyl; Cnaphalocrocis medinalis, Cmed; Helicoverpa armigera, Harm2; Ostrinia nubilalis, Onub; Helicoverpa zea, Hzea; Ostrinia furnacalis, Ofur; Hedya nubiferana, Hnub; Dendrolimus houi, Dhou; Mythimna separata, Msep; Sesamia inferens, Sinf; Agrotis segetum, Aseg.* (B) The tissue-specific expression of ORs in *M. troglodyta.* A: antenna; H: head; C: cuticle; M: midgut; F: forewing; Hi: hindwing.

Through the comparative analysis of the antennal transcriptome data of *M. troglodyta*, 74 OR genes were identified. After obtaining the sequences of these genes, the ORFs of these genes were searched and determined by ORFfinder. The results showed that 11 of them had incomplete ORFs. In addition, 25 sequences were found to coincide with the others of the 49 sequences by amino acid sequence alignment. Therefore, only 49 sequences with differences were selected to construct the phylogenetic tree. Through the phylogenetic tree, these OR genes were scattered in different branches. We found that *MtroOR38* clustered with other insect Orco genes. According to the high conservation of Orco sequences among different insect species, *MtroOR38* was identified as an Orco. In addition, 8 genes, such as *MtroOR48*, were clustered with pheromone receptors (PRs), and these genes were identified as PR (Fig. 3A, Supp Table S1 [online only]).

Eight OR genes with high expression in the transcriptome were selected from the identified OR genes, and their tissue expression profiles were measured by qRT–PCR. From the results, we found that among the 8 OR genes, 6 genes, *MtroOR1* (*F* = 99.447, df = 5,18, *P* < 0.0001), *MtroOR7* (*F* = 671.81, df = 5,18, *P* < 0.0001), *MtroOR13* (*F* = 598.52, df = 5,18, *P* < 0.0001), *MtroOR19* (*F* = 89.793, df = 5,18, *P* < 0.0001), *MtroOR27* (*F* = 294.41, df = 5,18, *P* < 0.0001), and *MtroOR29* (*F* = 832.83, df = 5,18, *P* < 0.0001), were highly expressed in the antennae. Among them, the expression of the *MtroOR13* gene in antennae and head was very similar, and there was no significant difference. The relative expression of the other two OR genes, *MtroOR10* (*F* = 586.3, df = 5,18, *P* < 0.0001) and *MtroOR16* (*F* = 132.8, df = 5,18, *P* < 0.0001), in the head was significantly higher than that in the antennae (Fig. 3B).

### Identification and Tissue Expression Profiles of Putative Odorant Binding Protein (OBP) Genes from *M. troglodyta*

**Fig. 4.**
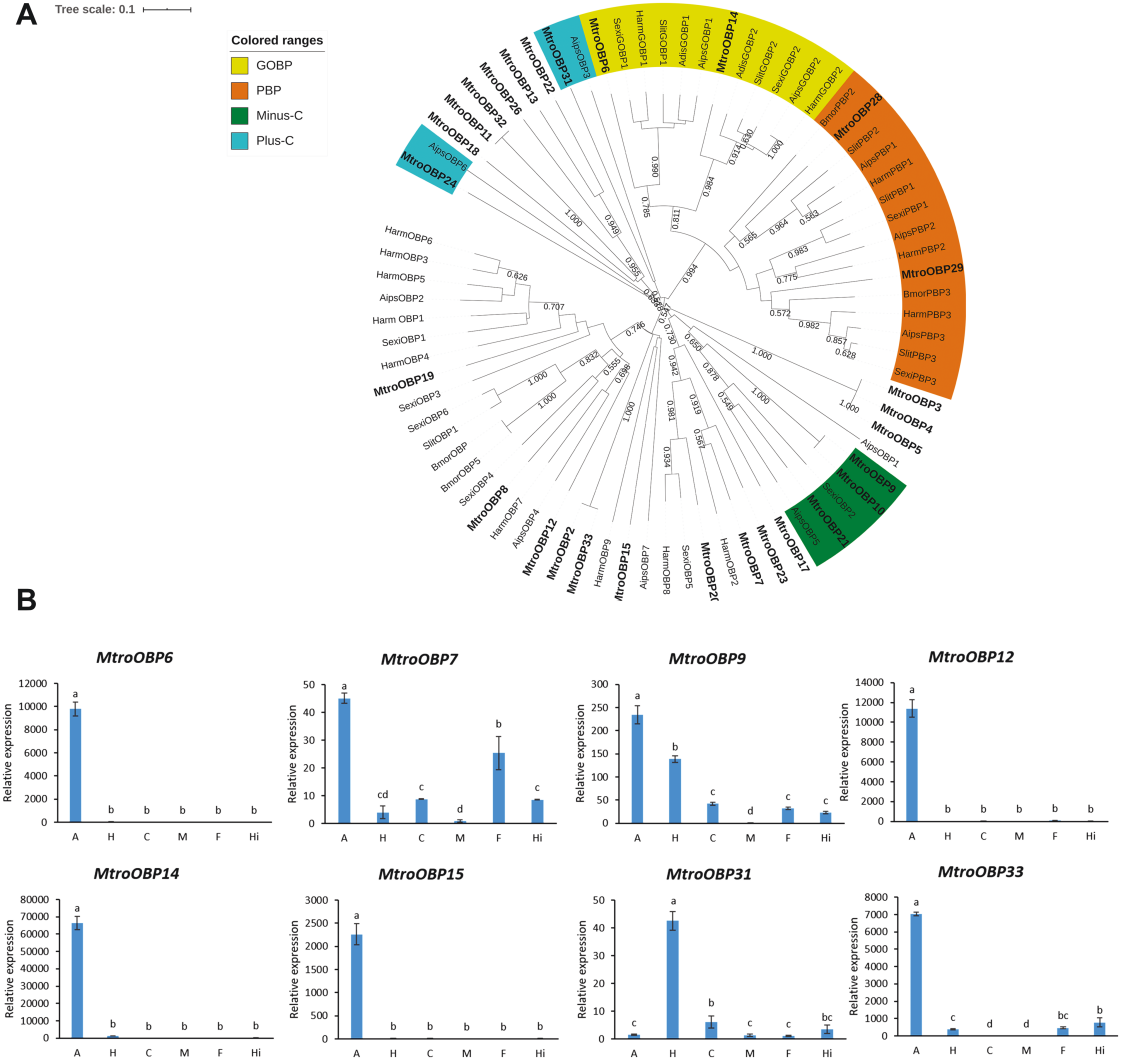
Phylogenetic analysis of OBPs and the tissue-specific expression of OBPs in *M. troglodyta*. (A) The phylogenetic tree was generated using MEGA X on the basis of ClustalW alignments. Branch numbers represent bootstrap values (1,000 replicates). *Bombyx mori, Bmor; Athetis dissimilis, Adis; Spodoptera litura, Slit; Spodoptera exigua, Sexi; Agrotis ipsilon, Aips; Helicoverpa armigera, Harm.* (B) The tissue-specific expression of OBPs in *M. troglodyte.* A: antenna; H: head; C: cuticle; M: midgut; F: forewing; Hi: hindwing.

OBPs have a conserved cysteine pattern of C1-X_25–30_-C2-X_3_-C3-X_36–42_-C4-X_8–14_-C5-X_8_-C6 (Xu et al. 2009). After detailed analysis of the assembled transcriptome and sequences alignment, 32 candidate OBPs were finally identified. However, during sequences alignment, we found that most candidate OBP sequences are less similar to known Lepidopteran OBPs, which may be due to the low sequences conservation among OBPs in different families. Among 32 candidate OBPs sequences, 31 candidate OBPs contained a complete ORF. In addition, 4 sequences were found to coincide with the other 28 sequences by amino acid sequence alignment. Therefore, only 28 sequences with differences were selected to construct the phylogenetic tree. According to the phylogenetic tree, these OBPs were clustered with different subfamilies, of which *MtroOBP6* and *MtroOBP14* aggregated with the identified GOBP. *MtroOBP28* and *MtroOBP29* were classed into a subgroup in the phylogenetic tree with previously characterized PBP. In addition, *MtroOBP9*, *MtroOBP10*, and *MtroOBP21* were distributed in the Minus-C subfamily, and *MtroOBP31* and *MtroOBP24* were distributed in the Plus-C subfamily (Fig. 4A, Supp Table S2 [online only]).

Eight of the identified OBP genes with high expression in the transcriptome were also selected for qRT–PCR to determine the tissue expression profile. For 5 of these genes, *MtroOBP6* (*F* = 1095.7, df = 5,18, *P* < 0.0001), *MtroOBP12* (*F* = 681.25, df = 5,18, *P* < 0.0001), *MtroOBP14* (*F* = 1188.3, df = 5,18, *P* < 0.0001), *MtroOBP15* (*F* = 406.01, df = 5,18, *P* < 0.0001), and *MtroOBP33* (*F* = 2268.9, df = 5,18, *P* < 0.0001), the relative expression in the antennae was extremely high, and almost no expression in other tissues. *MtroOBP7* (*F* = 154.12, df = 5,18, *P* < 0.0001) had the highest relative expression in the antennae, followed by in the forewings. In addition to the high expression level in the antennae, *MtroOBP9* (*F* = 418.06, df = 5,18, *P* < 0.0001) also had a high expression level in the head. *MtroOBP31* (*F* = 346, df = 5,18, *P* < 0.0001) was different from other genes, only highly expressed in the head, and the relative expression in other tissues was very low (Fig. 4B).

### Identification and Tissue Expression Profiles of Putative Chemosensory Protein (CSP) Genes from *M. troglodyta*

**Fig. 5.**
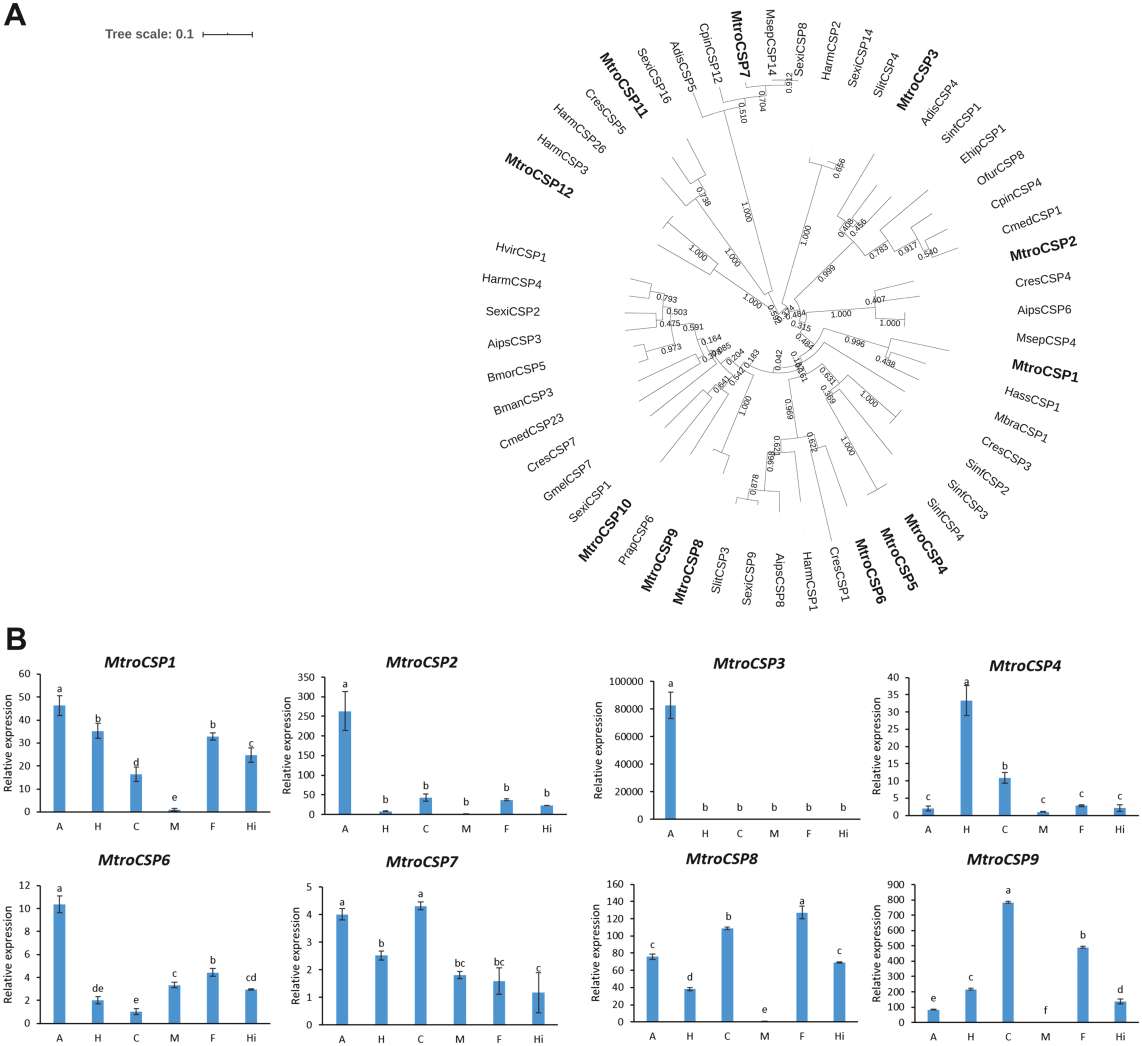
Phylogenetic analysis of CSPs and the tissue-specific expression of CSPs in *M. troglodyta*. (A) The phylogenetic tree was generated using MEGA X on the basis of ClustalW alignments. Branch numbers represent bootstrap values (1,000 replicates). *Helicoverpa assulta, Hass; Mamestra brassicae, Mbra; Clostera restitura, Cres; Agrotis ipsilon, Aips; Mythimna separata, Msep; Athetis dissimilis, Adis; Sesamia inferens, Sinf; Ostrinia furnacalis, Ofur; Conogethes pinicolalis, Cpin; Cnaphalocrocis medinalis, Cmed; Eogystia hippophaecolus, Ehip; Spodoptera exigua, Sexi; Spodoptera litura, Slit; Helicoverpa armigera, Harm; Heliothis virescens, Hvir; Bombyx mandarina, Bman; Bombyx mori, Bmor; Galleria mellonella, Gmel; Pieris rapae, Prap.* (B) The tissue-specific expression of CSPs in *M. troglodyta.* A: antenna; H: head; C: cuticle; M: midgut; F: forewing; Hi: hindwing.

Similar to OBPs, the CSPs have a conserved cysteine pattern of C1-X_6-8_-C2-X_16-21_-C3-X_2_-C4-X_3_ (Xu et al. 2009). Through sequence alignment and bioinformatics analysis, we obtained 13 putative CSPs from the transcriptome. Except for one sequence (*MtroCSP13*), the remaining 12 all contained the complete ORF. The sequences with complete ORF were selected to construct the phylogenetic tree. According to the sequence alignment results, we could see that the CSPs identified had higher similarities with the CSPs identified in Lepidoptera, which also implied that the CSPs sequence was more conservative. The phylogenetic tree also showed that these CSP genes were clustered together with other insect CSPs and distributed in different branches (Fig. 5A, Supp Table S3 [online only]).

We selected 8 genes with high expression in the transcriptome for subsequent tissue difference analysis. The tissue expression profiles of eight CSP genes showed that *MtroCSP1* (*F* = 88.30, df = 5,18, *P* < 0.0001), *MtroCSP2* (*F* = 7.0671, df = 5,18, *P* < 0.0001), *MtroCSP3* (*F* = 216.82, df = 5,18, *P* < 0.0001), and *MtroCSP6* (*F* = 221.94, df = 5,18, *P* < 0.0001) were highly expressed in the antennae, which was significantly different from other tissues. *MtroCSP4* (*F* = 126.69, df = 5,18, *P* < 0.0001) was the most expressed in the head, not the antennae. *MtroCSP7* (*F* = 36.228, df = 5,18, *P* < 0.0001) had a certain amount of expression in various tissues, the highest expression in antennae and cuticle, and there was no difference. The other two CSP genes, *MtroCSP8* (*F* = 557.24, df = 5,18, *P* < 0.0001)*, MtroCSP9* (*F* = 4928.8, df = 5,18, *P* < 0.0001), had a certain amount of expression in other tissues except for in the midguts (Fig. 5B).

### Identification and Tissue Expression Profiles of Putative Ionotropic Receptor (IR) Genes from *M. troglodyta*

**Fig. 6.**
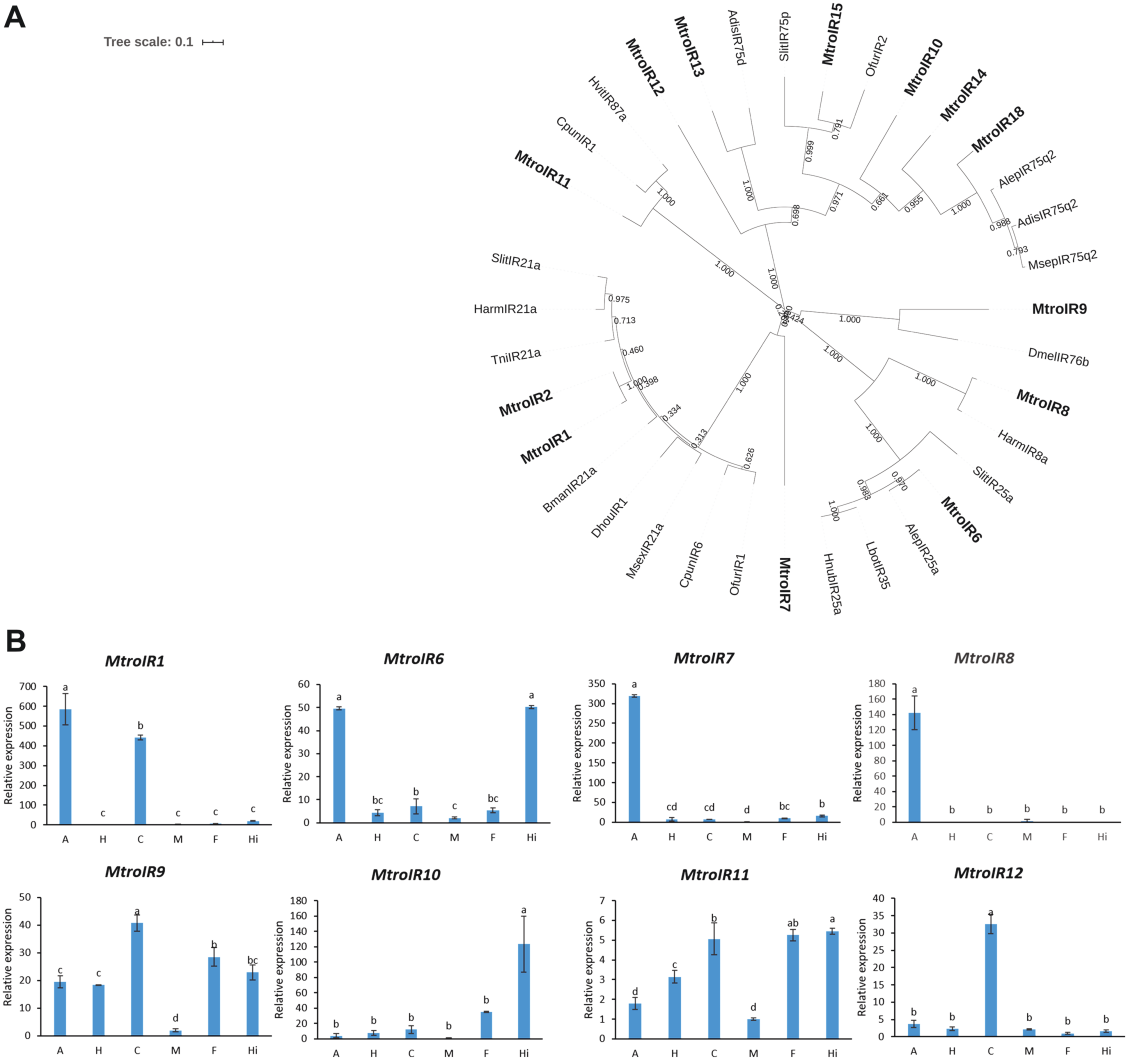
Phylogenetic analysis of IRs and the tissue-specific expression of IRs in *M. troglodyta*. (A) The phylogenetic tree was generated using MEGA X on the basis of ClustalW alignments. Branch numbers represent bootstrap values (1,000 replicates). *Athetis dissimilis, Adis; Bombyx mandarina, Bman; Conogethes punctiferalis, Cpun; Dendrolimus houi, Dhou; Drosophila melanogaster, Dmel; Hedya nubiferana, Hnub; Helicoverpa armigera, Harm; Heortia vitessoides, Hvit; Lobesia botrana, Lbot; Manduca sexta, Msex; Mythimna separata, Msep; Ostrinia furnacalis, Ofur; Spodoptera littoralis, Slit.Trichoplusia ni, Tni.* (B) The tissue-specific expression of IRs in *M. troglodyta*. A: antenna; H: head; C: cuticle; M: midgut; F: forewing; Hi: hindwing.

In total, 20 IR genes were identified from the antennal transcriptome. Sequence alignment results showed that most of them had high identity with other insect IR genes. However, 7 gene sequences were short, and it was speculated that they might be partial rather than full length, so they were not included in phylogenetic trees. The results of the phylogenetic tree showed that all IR genes were clustered with IR genes of other insects, of which *MtroIR1* and *MtroIR2* were clustered with genes of the IR21a family, indicating that these two genes might belong to the IR21a family. *MtroIR6* was clustered with the genes of the IR25a family, which means that IR6 may be a member of the IR25a family (Fig. 6A, Supp Table S4 [online only]).

We selected 8 IR genes with high expression in the transcriptome and verified the tissue expression profiles of these genes by qRT–PCR. The results showed that the tissue expression patterns of the eight IR genes were different. *MtroIR8* (*F* = 126.18, df = 5,18, *P* < 0.0001) was only expressed in the antenna and midgut. The expression level of *MtroIR1* (*F* = 219.32, df = 5,18, *P* < 0.0001) was the highest in the antennae, followed by the cuticle. The remaining 6 IR genes were expressed in various tissues, and the tissues with the highest relative expression levels were different. Among them, *MtroIR7* (*F* = 5689.6, df = 5,18, *P* < 0.0001) was only highly expressed in antennae, and *MtroIR6* (*F* = 718.68, df = 5,18, *P* < 0.0001) was highly expressed in antennae and hind wings, and *MtroIR10* (*F* = 31.571, df = 5,18, *P* < 0.0001) only had the highest relative expression in the hindwings, and *MtroIR12* (*F* = 318.67, df = 5,18, *P* < 0.0001) was only highly expressed in the cuticle (Fig. 6B).

### Identification and Tissue Expression Profiles of Putative Sensory Neuron Membrane Protein (SNMP) Genes from *M. troglodyta*

**Fig. 7.**
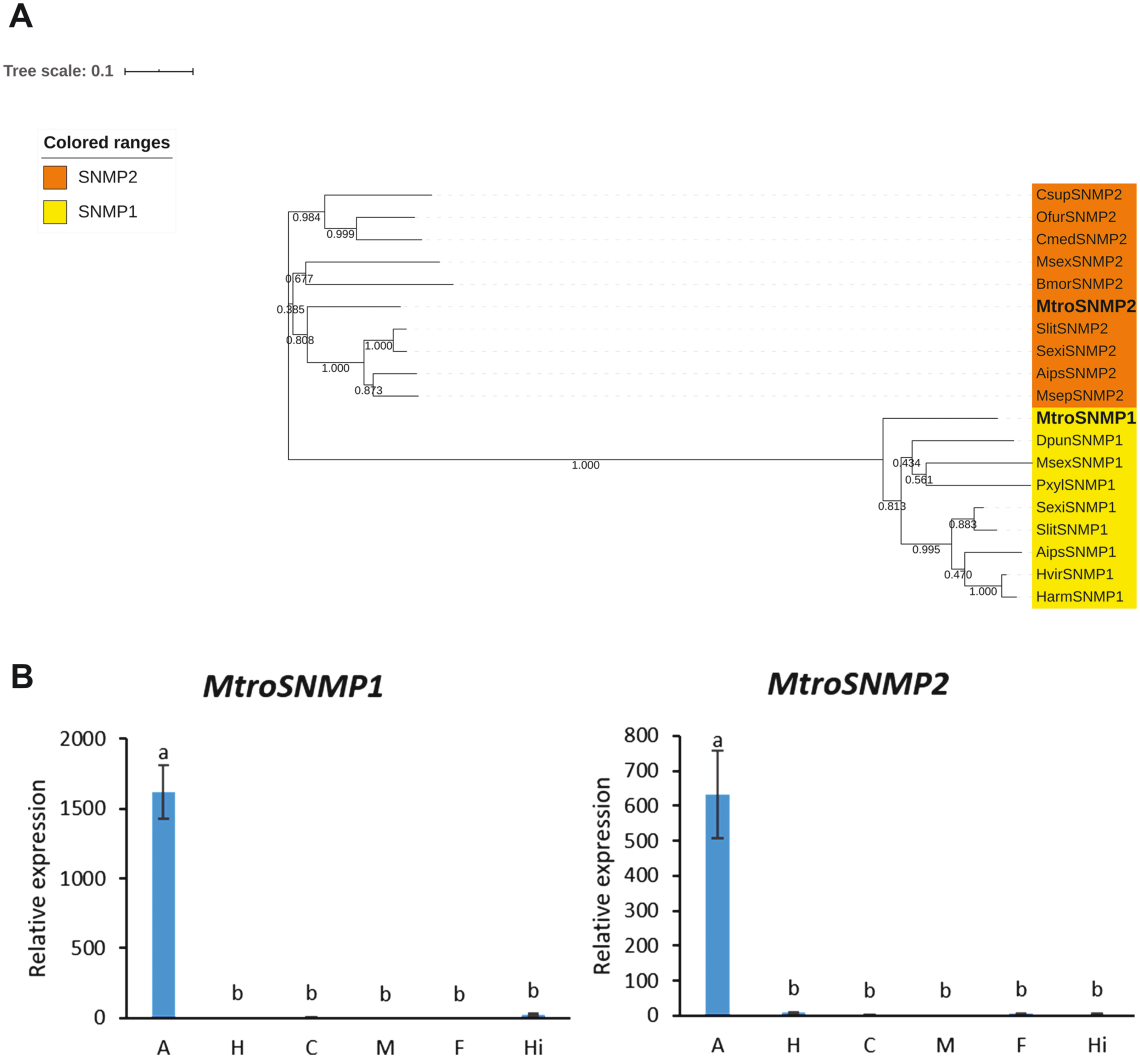
Phylogenetic analysis of SNMPs and the tissue-specific expression of SNMPs in *M. troglodyta*. (A) The phylogenetic tree was generated using MEGA X on the basis of ClustalW alignments. Branch numbers represent bootstrap values (1,000 replicates). *Agrotis ipsilon, Aips; Bombyx mori, Bmor; Chilo suppressalis, Csup; Cnaphalocrocis medinalis, Cmed; Dendrolimus punctatus, Dpun; Helicoverpa armigera, Harm; Heliothis virescens, Hvir; Manduca sexta, Msex; Mythimna separata, Msep; Ostrinia furnacalis, Ofur; Plutella xylostella, Pxyl; Spodoptera exigua, Sexi; Spodoptera litura, Slit.* (B) The tissue-specific expression of SNMPs in *M. troglodyta.* A: antenna; H: head; C: cuticle; M: midgut; F: forewing; Hi: hindwing..

In total, 3 SNMP genes were identified from the antennae transcription of *M. troglodyta*. After sequence alignment, it was found that *MtroSNMP1* and *MtroSNMP2* had complete ORFs, while *MtroSNMP3* had an incomplete ORF and the sequence length was too short. The sequences with complete ORFs were selected to construct the phylogenetic tree. The phylogenetic tree results showed that *MtroSNMP1* and *MtroSNMP2* were divided into two subfamilies, and clustered with the SNMP genes of the corresponding subfamilies. It indicated that *MtroSNMP1* belonged to the SNMP1 subfamily, and *MtroSNMP2* belonged to the SNMP2 subfamily (Fig. 7A, Supp Table S5 [online only]).

The tissue expression patterns of *MtroSNMP1* (*F* = 249.53, df = 5,18, *P* < 0.0001) and *MtroSNMP2* (*F* = 75.837, df = 5,18, *P* < 0.0001) were measured by qRT–PCR. The results showed that both *MtroSNMP1* and *MtroSNMP2* were expressed in various tissues, but the highest relative expression was in the antennae (Fig. 7B).

## Discussion

In recent years, with the development of molecular biology technology, many antennal transcriptomes of lepidoptera insects have been reported, such as *Helicoverpa armigera* (Hübner) (Liu et al., 2012), *Chilo suppressalis* (Walker) (Cao et al., 2014), *Manduca sexta* (L.) (Grosse-Wilde et al., 2011), *Epiphyas postvittana* (Walker) (Corcoran et al., 2015), *Cydia pomonella* (L.) (Bengtsson et al., 2012), *Dendrolimus houi* Lajonquière and *Dendrolimus kikuchii* Matsumura (Zhang et al., 2014), *Dioryctria abietella* (Denis & Schiffermüller) (Xing et al., 2021), *Athetis dissimilis (*Hampson) (Song et al., 2021), and *Spodoptera littoralis* Boisduval (Koutroumpa et al., 2021). In these reports, a large number of olfactory-related genes were identified. However, no olfactory-related genes in *M. troglodyta* have been reported. Therefore, this study used the Illumina HiSeq 2000 platform to sequence the transcriptome of the antennae of *M. troglodyta*. Through detailed analysis of the obtained transcriptome, we mined the olfactory-related genes to pave the way for exploring the olfactory mechanism of related species and to provide a basis for exploring prevention and treatment strategies with ORs as targets.

The results of the transcriptome data indicated that the quality and depth of sequencing was high at the transcriptome level. The average length of the antennal transcriptome was 1,092 bp. The length of the transcriptome varied in different Lepidoptera insects, such as *M. sexta* (460 bp) (Grosse-Wilde et al., 2011), *S. litura* (603 bp) (Feng et al., 2015), *Hyphantria cunea* Drury (829 bp) (Zhang et al.,2016), *Agrotis ipsilon* (Hufnagel) (967 bp) (Gu et al., 2014), *H. armigera* (991 bp) (Liu et al.,2012), *C. punctiferalis* (Guenée) (1144 bp) (Jia et al., 2016), *Streltzoviella insularis* (Staudinger) (1359 bp) (Yang et al., 2019), and *Carposina sasakii* Matsumura (1449 bp) (Tian et al., 2018). Functional database annotation results showed that 21.28% of unigenes (8,790) were annotated into COG, and only 6.83% of unigenes (2,822) were annotated into GO. This implied that there are still many new potential genes in the transcriptome, which should also include olfactory-related genes.

Through careful analysis of transcriptome data and sequence alignment, 142 olfactory genes were identified, including 74 odorant receptors (ORs), 32 odorant-binding proteins (OBPs), 13 chemosensory proteins (CSPs), 20 ionotropic receptors (IRs), and 3 sensory neuron membrane proteins (SNMPs). Our transcriptomic strategy appeared to be very efficient in identifying large sets of olfactory genes. For comparison, 47 ORs, 12 IRs, 26 OBPs, 12 CSPs, and 2 SNMPs were identified in *H. armigera* (Liu et al., 2012); 30 OBPs, 52 ORs, 17 CSPs, 14 IRs, 9 GRs, and 2 SNMPs were obtained in the *H. cunea* (Zhang et al., 2016); 47 ORs, 26 OBPs, 21 CSPs, 20 IRs, and 2 SNMPs were predicted in *C. suppressalis* (Cao et al., 2014). With the deepening of research and technological development, an increasing number of olfactory-related genes will be identified. For example, 32 OBPs, 16 CSPs, 70 ORs, 8 IRs, 1 GR, and 2 SNMPs were identified in *Mythimna separate* (Walker) (Chang et al., 2017), but it has been reported that the number of OBPs may be incomplete (Shiojiri et al. 2006).

Insect odorant receptors are highly differentiated members of the multigene family (Vosshall et al. 1999). At present, they are mainly divided into conventional odorant receptors and Orco (OR83b) (Pelletier et al. 2010). The insect odorant receptor family is very large. Since the first odorant receptor was identified in *D. melanogaster* (Pennisi, 1999) in 1999, a large number of other odorant receptors have been identified, including 79 ORs in the *Anopheles gambiae* Giles genome and 48 ORs in the *B. mori* genome (Fox et al., 2001, Sakurai et al., 2004, Nakagawa et al., 2005, Wanner et al., 2007a). By analyzing these identified odorant receptor sequences, it was found that the conventional odorant receptors have very low homology among insect species, and the pheromone receptors (PRs) have some homology in Lepidoptera moth insects (Krieger et al. 2004, Nakagawa et al. 2005, Wanner et al. 2007a,b; Mitsuno et al. 2008, Miura et al. 2009, 2010; Patch et al. 2009). However, Orco (OR83b) is highly conserved among different insect species, and the amino acid sequence identity can reach more than 70% (Jones et al. 2005, Yin et al. 2013). From the antennal transcriptome we identified a total of 74 ORs, of which 49 contained a complete ORF. The NJ phylogenetic tree constructed with OR sequences from other insects showed that *MtroOR38* was clustered in one branch with *PxylOR83b*, *HarmOR83b*, and *HzeaOR83b*. Additionally, according to the unified nomenclature system proposed by Vosshall (Vosshall et al. 2011), we considered *MtroOR38* to be a conservative Orco in *M. troglodyta*. We also found that 8 genes, including *MtroOR48,* aggregated with the genes of the PR family. These 8 genes of *M. troglodyta* were presumed to belong to the PR family by sequence comparison, and may play a role in detecting pheromones, especially sex pheromones. We studied the tissue expression patterns of ORs by qRT–PCR. As with most reports, a large number of ORs were mainly expressed in the antennae. In particular, the relative expression of *MtroOR10* and *MtroOR16* in the head was higher than that in the antennae. It was speculated that the mouthparts also had the distribution of olfactory sensors (Malpel et al. 2008). This finding suggested that *MtroOR10* and *MtroOR16* may be involved in other physiological activities. Among other insects, such as *S. litura*, *SlitOR2* was slightly expressed in the head (Brigaud et al. 2009), and *A. gambia* (Pitts et al., 2004) and *Spodoptera frugiperda* (Smith) (Krieger et al., 2002) also have a small amount of *OR2* expression. Therefore, further functional research on *MtroOR* will help to better understand the mechanism of *M. troglodyta* recognition of external odors at the OR level.

OBPs are important participants in insect olfactory behavior, and the combination with the odorant in the environment is also the first step to start odorant perception. Then, these odorants are transported to ORs through the lymph. (Vogt et al. 1999, Leal 2005). Based on the antennal transcriptome, we obtained 32 OBPs through homology alignment. Through the NJ phylogenetic tree constructed with other insect OBPs, we found that these OBPs were divided into several different branches. *MtroOBP6* and *MtroOBP14* were presumed to belong to the GOBP subfamily and were also related to GOBP1 and GOBP2 in *M. troglodyta*, respectively. According to the number of conserved cysteines, OBPs are divided into different subfamilies, which means that their molecular structures are different. This also implies that these OBPs may also participate in other life activities in addition to participating in olfactory mechanisms. There have been many reports that OBPs are highly expressed in insect antennae. We also studied the tissue expression patterns of OBPs by qRT–PCR. Most of these OBPs are abundantly expressed in antennae, indicating that these genes have a specific role in the olfactory process. In addition, there have been reports that OBPs are also expressed in other parts of the insect body and at different growth stages. Similar results were found in our study; e.g., although *MtroOBP9* has the highest relative expression in the antennae, it also has a high expression in the head. It is also worth noting that *MtroOBP7* has the highest expression in the antennae, but it also has a high expression in the forewings. Another gene, *MtroOBP31*, is only highly expressed in the head, which is significantly different from the relative expression in the antennae. This indicates that insect OBP exists in other tissues (wings) in addition to antennae, which may be related to adapting to complex olfactory activities. It has been reported that in *S. litura* (Gu et al., 2015), 3 OBPs showed body-specific expression, and 18 OBPs had similar expression levels in the antennae and other body tissues.

In total, 13 CSPs were identified, of which 12 CSPs had complete ORFs. Insect CSPs can identify nonvolatile chemicals in the environment and they are a class of acidic soluble proteins. CSP also has a structural pattern similar to OBP, but the difference is that CSP has only 4 conserved cysteines (Xu et al. 2009). It is more conservative than OBPs. Our NJ phylogenetic tree and sequence alignment have also proved that CSPs have high sequence homology. In addition, the expression profile of CSP in *B. mori* was different at different developmental stages, and it was expressed in multiple tissues, which indicates that CSP bears many complex chemosensory functions (Gong et al. 2007). Previous studies have shown that antennae are not the only tissue for CSP gene expression, and the tissue expression profiles of these genes are relatively diverse. However, our results showed that *MtroCSP2* and *MtroCSP3* are antennae-specifically expressed, which suggests that these two genes play an important role in the olfactory process. The differential expression of other genes also precisely reflected the diversity of CSP gene function. This suggests that they may also have chemical sensing and other nonolfactory effects.

Additionally, proteins such as IRs, SNMPs, and GRs are also involved in the olfactory process of insects and have been identified in our antennal transcriptome, with the exception of GRs. The reason why GRs have not been identified may be that GRs are mostly expressed in the taste organs of insects, and the expression amount in the antennae is very small. We also identified 20 IRs and 3 SNMPs and constructed NJ phylogenetic trees using sequences containing complete ORFs. IRs are a class of conservative chemosensory receptors. The study of such genes has been relatively recent. Benton identified IRs in the *D. melanogaster* genome for the first time in 2009 (Benton et al. 2009). It was found that IRs of different tissues might play different functions in *Drosophila melanogaster* (Ai et al., 2013). Neurons expressing *IR84a* in antennae participated in the process of Drosophila looking for food and determining spawning sites (Dahanukar et al. 2005). Croset et al. (2010) found that *DmilIR8a* was mainly highly expressed in the antennae of *Drosophila*, suggesting that it might be involved in the function of olfactory recognition. In our experiment, *MtroIR1*, *MtroIR 6*, *MtroIR 7*, and *MtroIR 8* were also highly expressed in antennae, so it was speculated that they might also be involved in olfactory recognition. Recent research results show that IRs play an important role in *Drosophila’s* perception of environmental temperature changes (Ni et al. 2016). IR expressed in dorsal organ cool cells of *Drosophila* was involved in sensing temperature changes in the environment (Klein et al. 2015). Therefore, we speculated that the high expression of IR in the cuticle might also be related to the perception of temperature changes. SNMPs are specific proteins on olfactory neurons and dendritic membranes (Rogers et al. 2001a), with two subfamilies. The two SNMPs in our transcriptome belonged to the SNMP1 and SNMP2 subfamilies. Many studies have shown that SNMPs are related to insect odorant recognition, but there are still many speculative functions that need to be further studied (Rogers et al. 2001b). The tissue expression patterns showed that these two kinds of SNMP were expressed almost exclusively in the antennae, which just proved that they played an important role in the olfactory process of *M. troglodyta* and was consistent with the previous results.

Our study constructed the transcriptome of the *M. troglodyta* antennae, and identified 142 olfactory-related genes. The tissue-specific expression patterns of these genes were analyzed by qRT–PCR, and most of them were expressed in large numbers in the antennae. These results lay the foundation for further research on the olfactory-related proteins of *M. troglodyta* and provide a theoretical basis for a new approach to controlling *M. troglodyta*. For example, studying odorant receptors and combining them with RNA interference, could increase the sensitivity of insects to pesticides. Furthermore, it also provides a research foundation for the development of new, efficient, and environmentally friendly attractants or repellents.

## Supplementary Data

Supplementary data are available at *Journal of Insect Science* online.

Fig. S1. Nr classification of all *M. troglodyta* unigenes.

Table S1. OR genes used in phylogenetic tree.

Table S2. OBP genes used in phylogenetic tree.

Table S3. CSP genes used in phylogenetic tree.

Table S4. IR genes used in phylogenetic tree.

Table S5. SNMP genes used in phylogenetic tree.

ieac056_suppl_Supplementary_Figure_S1

ieac056_suppl_Supplementary_Table_S1

ieac056_suppl_Supplementary_Table_S2

ieac056_suppl_Supplementary_Table_S3

ieac056_suppl_Supplementary_Table_S4

ieac056_suppl_Supplementary_Table_S5
