# Supplementary figures and images for: Analysis of the Antennal Transcriptome and Identification of Tissue-specific Expression of Olfactory-related Genes in Micromelalopha troglodyta (Lepidoptera: Notodontidae)
Source: J Insect Sci. 2022 Sep 27;22(5):8. doi: 10.1093/jisesa/ieac056 (PMC9513789; doi:10.1093/jisesa/ieac056)

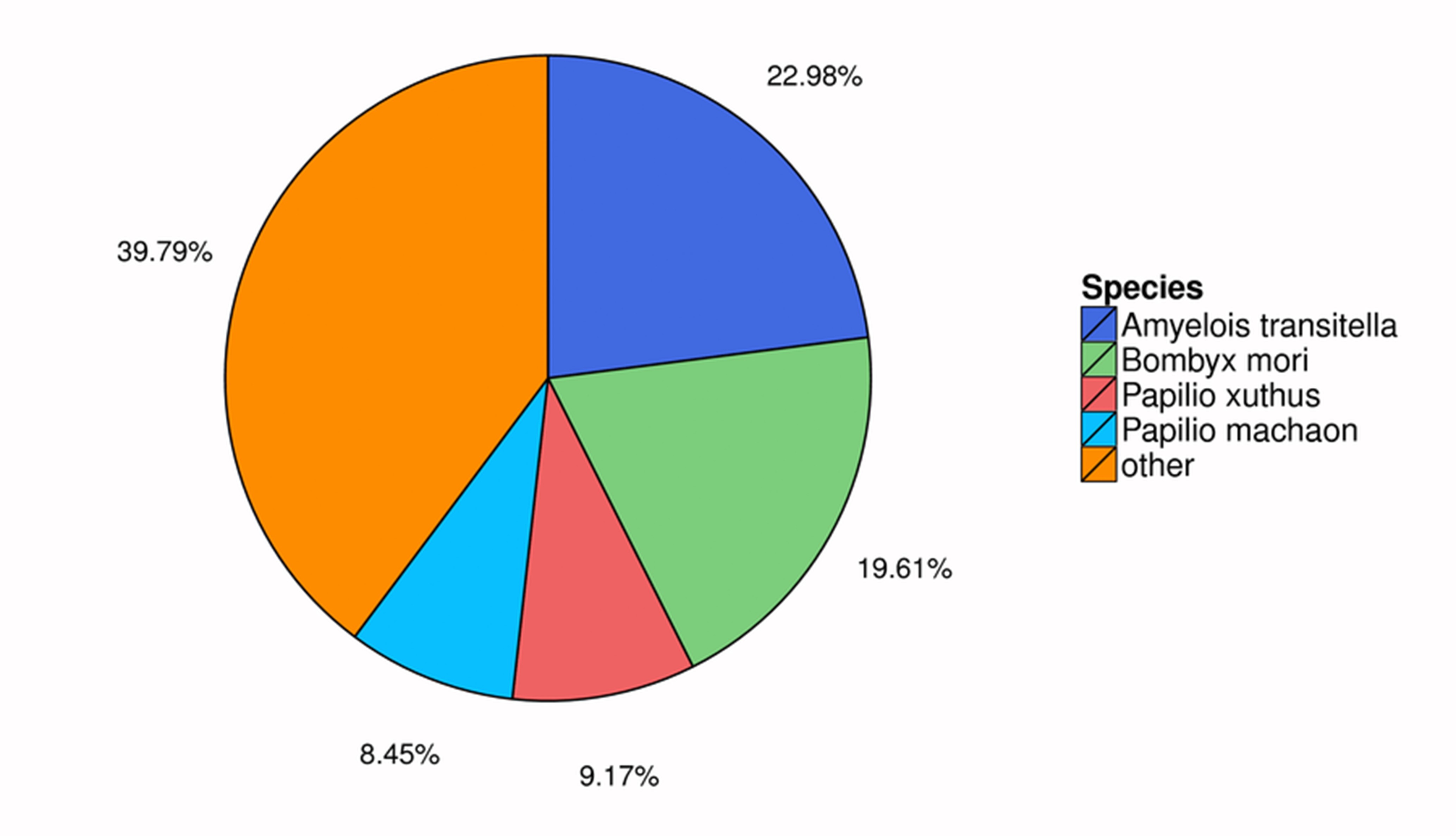

Supplement: ieac056_suppl_Supplementary_Figure_S1 [file ieac056_suppl_supplementary_figure_s1.jpeg]
