# Supplementary material for: Analysis of the Antennal Transcriptome and Identification of Tissue-specific Expression of Olfactory-related Genes in Micromelalopha troglodyta (Lepidoptera: Notodontidae)
Source: J Insect Sci. 2022 Sep 27;22(5):8. doi: 10.1093/jisesa/ieac056 (PMC9513789; doi:10.1093/jisesa/ieac056)
Supplement: ieac056_suppl_Supplementary_Table_S1 [file ieac056_suppl_supplementary_table_s1.docx]

Table S1 OR genes and their accession number used in phylogenetic tree

| Species | Gene name | Accession No. |
| --- | --- | --- |
| *Bombyx mori* | *BmorOR1* | NP_001036875.1 |
| *Bombyx mori* | *BmorOR3* | NP_001036925.1 |
| *Bombyx mori* | *BmorOR4* | NP_001036926.1 |
| *Bombyx mori* | *BmorOR5* | NP_001036927.1 |
| *Bombyx mori* | *BmorOR7* | NP_001106227.1 |
| *Bombyx mori* | *BmorOR12* | NP_001104829.1 |
| *Bombyx mori* | *BmorOR15* | NP_001091789.1 |
| *Dendrolimus kikuchii* | *DkikOR33* | KF487712.1 |
| *Dendrolimus kikuchii* | *DkikOR28* | KF487707.1 |
| *Manduca sexta* | *MsexOR1* | CUQ99387.1 |
| *Manduca sexta* | *MsexOR3* | ACM18061.1 |
| *Manduca sexta* | *MsexOR4* | ADM32897.1 |
| *Manduca sexta* | *MsexOR5* | CUQ99389.1 |
| *Manduca sexta* | *MsexOR13* | CUQ99397.1 |
| *Manduca sexta* | *MsexOR15* | CUQ99398.1 |
| *Manduca sexta* | *MsexOR29* | CUQ99410.1 |
| *Manduca sexta* | *MsexOR17* | CUQ99400.1 |
| *Manduca sexta* | *MsexOR50* | AFL70813.1 |
| *Manduca sexta* | *MsexOR51* | AFL70814.1 |
| *Spodoptera litura* | *SlitOR12* | JX999588.1 |
| *Spodoptera litura* | *SlitOR16* | EU979117.1 |
| *Spodoptera litura* | *SlitOR51* | JX999586.1 |
| *Athetis dissimilis* | *AdisOR28* | KR935725.1 |
| *Athetis dissimilis* | *AdisOR62* | KR935753.1 |
| *Athetis dissimilis* | *AdisOR45* | KR935743.1 |
| *Helicoverpa assulta* | *HassOR44* | KJ542694.1 |
| *Athetis lepigone* | *AlepOR8* | KT588103.1 |
| *Athetis lepigone* | *AlepOR20* | KT588115.1 |
| *Plutella xylostella* | *PxylOR83b* | NM_001309102.1 |
| *Cnaphalocrocis medinalis* | *CmedPR1* | ALT31681.1 |
| *Cnaphalocrocis medinalis* | *CmedPR2* | ALT31682.1 |
| *Cnaphalocrocis medinalis* | *CmedPR3* | ALT31683.1 |
| *Cnaphalocrocis medinalis* | *CmedPR4* | ALT31684.1 |
| *Helicoverpa armigera* | *HarmPR14.2* | AJG42377.1 |
| *Helicoverpa armigera* | *HarmOR83b* | HQ186284.1 |
| *Ostrinia nubilalis* | *OnubOR1* | AIZ94618.1 |
| *Ostrinia nubilalis* | *OnubOR3* | AIZ94617.1 |
| *Ostrinia nubilalis* | *OnubOR5* | AIZ94615.1 |
| *Ostrinia nubilalis* | *OnubOR6* | AIZ94614.1 |
| *Helicoverpa zea* | *HzeaOr83b* | AY843204.1 |
| *Ostrinia furnacalis* | *OfurOR16* | LC002710.1 |
| *Hedya nubiferana* | *HnubOR63* | KY283626.1 |
| *Dendrolimus houi* | *DhouOR16* | KF487662.1 |
| *Mythimna separata* | *MsepOR1* | AB263110.1 |
| *Sesamia inferens* | *SinfOR* | KC960476.2 |
| *Agrotis segetum* | *AsegOR4* | KC526967.1 |
