# Supplementary material for: Analysis of the Antennal Transcriptome and Identification of Tissue-specific Expression of Olfactory-related Genes in Micromelalopha troglodyta (Lepidoptera: Notodontidae)
Source: J Insect Sci. 2022 Sep 27;22(5):8. doi: 10.1093/jisesa/ieac056 (PMC9513789; doi:10.1093/jisesa/ieac056)
Supplement: ieac056_suppl_Supplementary_Table_S2 [file ieac056_suppl_supplementary_table_s2.docx]

Table S2 OBP genes and their accession number used in phylogenetic tree

| Species | Gene name | Accession No. |
| --- | --- | --- |
| *Bombyx mori* | *BmorPBP3* | NM_001083626.1 |
| *Bombyx mori* | *BmorPBP2* | AM403100.1 |
| *Bombyx mori* | *BmorOBP5* | NM_001146717.1 |
| *Bombyx mori* | *BmorOBP* | AB525759.1 |
| *Athetis dissimilis* | *AdisGOBP1* | KR780027.1 |
| *Athetis dissimilis* | *AdisGOBP2* | KR780028.1 |
| *Spodoptera litura* | *SlitGOBP1* | KP331514.1 |
| *Spodoptera litura* | *SlitGOBP2* | KP331515.1 |
| *Spodoptera litura* | *SlitPBP1* | KP331511.1 |
| *Spodoptera litura* | *SlitPBP2* | KP331512.1 |
| *Spodoptera litura* | *SlitPBP3* | KP331513.1 |
| *Spodoptera litura* | *SlitOBP1* | KT261647.1 |
| *Spodoptera exigua* | *SexiGOBP1* | GU082319.1 |
| *Spodoptera exigua* | *SexiGOBP2* | AJ294808.1 |
| *Spodoptera exigua* | *SexiPBP1* | AY743351.1 |
| *Spodoptera exigua* | *SexiPBP3* | GU082320.1 |
| *Spodoptera exigua* | *SexiOBP1* | HQ234488.1 |
| *Spodoptera exigua* | *SexiOBP2* | HQ234489.1 |
| *Spodoptera exigua* | *SexiOBP3* | HQ234490.1 |
| *Spodoptera exigua* | *SexiOBP4* | HQ234491.1 |
| *Spodoptera exigua* | *SexiOBP5* | JQ905624.1 |
| *Spodoptera exigua* | *SexiOBP6* | JQ905625.1 |
| *Agrotis ipsilon* | *AipsGOBP1* | JQ822243.1 |
| *Agrotis ipsilon* | *AipsGOBP2* | JQ822244.1 |
| *Agrotis ipsilon* | *AipsPBP1* | JQ822240.1 |
| *Agrotis ipsilon* | *AipsPBP2* | JQ822241.1 |
| *Agrotis ipsilon* | *AipsPBP3* | JQ822242.1 |
| *Agrotis ipsilon* | *AipsOBP1* | JX863689.1 |
| *Agrotis ipsilon* | *AipsOBP2* | JX863690.1 |
| *Agrotis ipsilon* | *AipsOBP3* | JX863691.1 |
| *Agrotis ipsilon* | *AipsOBP4* | JX863692.1 |
| *Agrotis ipsilon* | *AipsOBP5* | JX863693.1 |
| *Agrotis ipsilon* | *AipsOBP6* | JX863694.1 |
| *Agrotis ipsilon* | *AipsOBP7* | JX863695.1 |
| *Helicoverpa armigera* | *HarmGOBP1* | AAL09821.1 |
| *Helicoverpa armigera* | *HarmGOBP2* | CAC08211.1 |
| *Helicoverpa armigera* | *HarmPBP1* | AEB54585.1 |
| *Helicoverpa armigera* | *HarmPBP2* | AEB54583.1 |
| *Helicoverpa armigera* | *HarmPBP3* | AAO16091.1 |
| *Helicoverpa armigera* | *Harm OBP1* | AEB54580.1 |
| *Helicoverpa armigera* | *Harm OBP2* | AEB54586.1 |
| *Helicoverpa armigera* | *Harm OBP3* | AEB54582.1 |
| *Helicoverpa armigera* | *Harm OBP4* | AEB54584.1 |
| *Helicoverpa armigera* | *Harm OBP5* | AEB54581.1 |
| *Helicoverpa armigera* | *Harm OBP6* | AEB54587.1 |
| *Helicoverpa armigera* | *Harm OBP7* | AEB54591.1 |
| *Helicoverpa armigera* | *Harm OBP8* | AEB54589.1 |
| *Helicoverpa armigera* | *Harm OBP9* | AEB54592.1 |
