# Supplementary material for: Analysis of the Antennal Transcriptome and Identification of Tissue-specific Expression of Olfactory-related Genes in Micromelalopha troglodyta (Lepidoptera: Notodontidae)
Source: J Insect Sci. 2022 Sep 27;22(5):8. doi: 10.1093/jisesa/ieac056 (PMC9513789; doi:10.1093/jisesa/ieac056)
Supplement: ieac056_suppl_Supplementary_Table_S3 [file ieac056_suppl_supplementary_table_s3.docx]

Table S3 CSP genes and their accession number used in phylogenetic tree

| Species | Gene name | Accession No. |
| --- | --- | --- |
| *Helicoverpa assulta* | *HassCSP1* | ABB91378.1 |
| *Mamestra brassicae* | *MbraCSP1* | AAF71289.1 |
| *Clostera restitura* | *CresCSP1* | AYC12346.1 |
| *Clostera restitura* | *CresCSP3* | AYC12348.1 |
| *Clostera restitura* | *CresCSP4* | AYC12349.1 |
| *Clostera restitura* | *CresCSP5* | AYC12350.1 |
| *Clostera restitura* | *CresCSP7* | AYC12352.1 |
| *Agrotis ipsilon* | *AipsCSP3* | AGR39573.1 |
| *Agrotis ipsilon* | *AipsCSP6* | AGR39576.1 |
| *Agrotis ipsilon* | *AipsCSP8* | AGR39578.1 |
| *Mythimna separate* | *MsepCSP4* | AWT22250.1 |
| *Mythimna separata* | *MsepCSP14* | AWT22248.1 |
| *Athetis dissimilis* | *AdisCSP4* | AND82446.1 |
| *Athetis dissimilis* | *AdisCSP5* | AND82447.1 |
| *Sesamia inferens* | *SinfCSP1* | AGY49270.1 |
| *Sesamia inferens* | *SinfCSP2* | AGY49265.1 |
| *Sesamia inferens* | *SinfCSP3* | AGY49266.1 |
| *Sesamia inferens* | *SinfCSP4* | AGY49262.1 |
| *Ostrinia furnacalis* | *OfurCSP8* | BAV56812.1 |
| *Conogethes pinicolalis* | *CpinCSP4* | QFR36131.1 |
| *Conogethes pinicolalis* | *CpinCSP12* | QFR36139.1 |
| *Cnaphalocrocis medinalis* | *CmedCSP1* | AIX97835.1 |
| *Cnaphalocrocis medinalis* | *CmedCSP23* | ALT31605.1 |
| *Eogystia hippophaecolus* | *EhipCSP1* | AOG12894.1 |
| *Spodoptera exigua* | *SexiCSP1* | ABM67688.1 |
| *Spodoptera exigua* | *SexiCSP2* | ABM67689.1 |
| *Spodoptera exigua* | *SexiCSP8* | AKT26485.1 |
| *Spodoptera exigua* | *SexiCSP9* | AVC68635.1 |
| *Spodoptera exigua* | *SexiCSP14* | AKT26490.1 |
| *Spodoptera exigua* | *SexiCSP16* | AKT26491.1 |
| *Spodoptera litura* | *SlitCSP3* | ALJ30214.1 |
| *Spodoptera litura* | *SlitCSP4* | ALJ30215.1 |
| *Helicoverpa armigera* | *HarmCSP1* | AIW65097.1 |
| *Helicoverpa armigera* | *HarmCSP2* | AIW65104.1 |
| *Helicoverpa armigera* | *HarmCSP4* | AEX07269.1 |
| *Helicoverpa armigera* | *HarmCSP3* | AIW65100.1 |
| *Helicoverpa armigera* | *HarmCSP26* | ASA40088.1 |
| *Heliothis virescens* | *HvirCSP1* | AAM77041.1 |
| *Bombyx mandarina* | *BmanCSP3* | ACA24124.1 |
| *Bombyx mori* | *BmorCSP5* | BAF34353.1 |
| *Galleria mellonella* | *GmelCSP7* | QEI46805.1 |
| *Pieris rapae* | *PrapCSP6* | QDW65472.1 |
