# Supplementary material for: Analysis of the Antennal Transcriptome and Identification of Tissue-specific Expression of Olfactory-related Genes in Micromelalopha troglodyta (Lepidoptera: Notodontidae)
Source: J Insect Sci. 2022 Sep 27;22(5):8. doi: 10.1093/jisesa/ieac056 (PMC9513789; doi:10.1093/jisesa/ieac056)
Supplement: ieac056_suppl_Supplementary_Table_S4 [file ieac056_suppl_supplementary_table_s4.docx]

Table S4 IR genes and their accession number used in phylogenetic tree

| Species | Gene name | Accession No. |
| --- | --- | --- |
| *Trichoplusia ni* | *Tni IR21a* | XP_026731589.1 |
| *Helicoverpa armigera* | *HarmIR21a* | XP_021200448.1 |
| *Helicoverpa armigera* | *HarmIR8a* | QBX91201.1 |
| *Spodoptera litura* | *SlitIR21a* | XP_022821953.1 |
| *Ostrinia furnacalis* | *OfurIR1* | BAR64797.1 |
| *Ostrinia furnacalis* | *OfurIR2* | BAR64805.1 |
| *Manduca sexta* | *MsexIR21a* | XP_030021838.1 |
| *Bombyx mandarina* | *BmanIR21a* | XP_028032585.1 |
| *Conogethes punctiferalis* | *CpunIR1* | ARO76464.1 |
| *Conogethes punctiferalis* | *CpunIR6* | ARO76469.1 |
| *Dendrolimus houi* | *DhouIR1* | AII01113.1 |
| *Athetis lepigone* | *AlepIR25a* | AOE47999.1 |
| *Spodoptera littoralis* | *SlitIR25a* | ADR64679.1 |
| *Lobesia botrana* | *LbotIR35* | AXF48866.1 |
| *Hedya nubiferana* | *HnubIR25a* | AST36228.1 |
| *Drosophila melanogaster* | *DmelIR76b* | NP_649176.1 |
| *Heortia vitessoides* | *HvitIR87a* | AZB49412.1 |
| *Athetis dissimilis* | *AdisIR75d* | ALM24944.1 |
| *Athetis dissimilis* | *AdisIR75q2* | ALM24940.1 |
| *Mythimna separata* | *MsepIR75q2* | ARB05668.1 |
| *Athetis lepigone* | *AlepIR75q2* | AOE47995.1 |
| *Spodoptera littoralis* | *SlitIR75p* | ADR64684.1 |
