# Supplementary material for: Analysis of the Antennal Transcriptome and Identification of Tissue-specific Expression of Olfactory-related Genes in Micromelalopha troglodyta (Lepidoptera: Notodontidae)
Source: J Insect Sci. 2022 Sep 27;22(5):8. doi: 10.1093/jisesa/ieac056 (PMC9513789; doi:10.1093/jisesa/ieac056)
Supplement: ieac056_suppl_Supplementary_Table_S5 [file ieac056_suppl_supplementary_table_s5.docx]

Table S5 SNMP genes and their accession number used in phylogenetic tree

| Species | | Gene name | | Accession No. |
| --- | --- | --- | --- | --- |
| *Manduca sexta* | | *MsexSNMP1* | AAG49366.1 | |
| *Plutella xylostella* | | *PxylSNMP1* | ADK66278.1 | |
| *Spodoptera exigua* | | *SexiSNMP1* | AGN52676.1 | |
| *Dendrolimus punctatus* | | *DpunSNMP1* | ARO70303.1 | |
| *Heliothis virescens* | *HvirSNMP1* | | CAB65739.1 | |
| *Agrotis ipsilon* | *AipsSNMP1* | | AGF87119.1 | |
| *Spodoptera litura* | *SlitSNMP1* | | XP_022834366.1 | |
| *Helicoverpa armigera* | *HarmSNMP1* | | Q8I9S1.1 | |
| *Spodoptera litura* | *SlitSNMP2* | | AGN48099.1 | |
| *Agrotis ipsilon* | | *AipsSNMP2* | AGF87120.1 | |
| *Manduca sexta* | *MsexSNMP2* | | AAG49365.1 | |
| *Chilo suppressalis* | *CsupSNMP2* | | AFS50074.1 | |
| *Bombyx mori* | | *BmorSNMP2* | XP_012547405.1 | |
| *Ostrinia furnacalis* | *OfurSNMP2* | | ADQ73891.1 | |
| *Cnaphalocrocis medinalis* | *CmedSNMP2* | | AFG73003.1 | |
| *Spodoptera exigua* | | *SexiSNMP2* | AGN52677.1 | |
| *Helicoverpa armigera* | *HarmSNMP2* | | XP_021184873.1 | |
| *Mythimna separata* | *MsepSNMP2* | | JAV45785.1 | |
